# Dissolved oxygen and nitrates gradient influence marine microbial complexity and stability in Beibu Gulf

**DOI:** 10.3389/fmicb.2025.1622150

**Published:** 2025-06-25

**Authors:** Qing He, Qingxiang Chen, Xinyi Qin, Shengyao Zhou, Rajapakshalage Thashikala Nethmini, Gonglingxia Jiang, Qinghua Hou, Xiaolei Li, Laizhen Huang, Ke Dong, Lingling Xie, Nan Li

**Affiliations:** ^1^Key Laboratory of Climate, Resources and Environment in Continental Shelf Sea and Deep Sea of Department of Education of Guangdong Province, Department of Oceanography, Key Laboratory for Coastal Ocean Variation and Disaster Prediction, College of Ocean and Meteorology, Guangdong Ocean University, Zhanjiang, China; ^2^Key Laboratory of Environment Change and Resources Use in Beibu Gulf, Ministry of Education (Nanning Normal University), Nanning, China; ^3^College of Environmental Science and Engineering, Guilin University of Technology, Guilin, China; ^4^Department of Biological Sciences, Kyonggi University, Suwon-si, Republic of Korea

**Keywords:** bacterial communities, co-occurrence network, community stability, community complexity, environmental threshold

## Abstract

Environmental gradients are important for bacteria community in marine ecosystems. However, the tipping points of environmental heterogeneity and ecological responses to disturbances in marine ecosystems are still unclear. In this study, we sampled seawater from different layers of Beibu Gulf to investigate bacterial composition, diversity, network complexity and stability, and environmental thresholds. Proteobacteria (40.38%), Cyanobacteria (27.35%), and Actinobacteria (18.24%) were dominant across all three layers. Alpha diversity was higher in the bottom layer (BL), and beta diversity were greater in the middle layer (ML). Deterministic processes significantly structured bacterial communities. The BL had the most complex network, while the ML showed the highest stability. Dissolved oxygen (DO) influenced bacterial dissimilarity and community stability, while NO_3_^−^ drives complexity. Segmented regression identified environmental stress thresholds: pH = 7.79, TN = 7.48 mg/L, and temperature = 27.9°C. DO thresholds for beta diversity were 6.31 mg/L, 6.25 mg/L and 5.93 mg/L across layers, and for βNTI were 6.57 mg/L and 6.24 mg/L in ML and BL. Tipping points for community stability occurred at DO levels of 6.71 mg/L, 5.80 mg/L and 5.94 mg/L. NO_3_^−^ thresholds of complexity appeared in the SL (at 0.003 mg/L) and BL (0.020 mg/L) samples, but not in ML. This study provides new insights into bacterial stress resistance and community maintenance in the subtropical Gulf marine environments.

## 1 Introduction

Community assembly and species coexistence of marine microorganisms play important and indispensable roles in marine ecosystems, serving as the cornerstone for maintaining ecological balance and contributing significantly to environmental sustainability (Jiao et al., 2020; Pan et al., 2022). Recent research has indicated that environmental factors significantly influence the assembly and coexistence of marine bacterial communities, driving microbial turnover and species diversity across diverse maritime regions (Lin et al., 2024; Niu et al., 2024). For instance, Li et al. (2021) found that the assembly processes of marine bacteria responds to the Chlorophyll *a* concentrations and N/P ratios in the Eastern Indian Ocean. Ren et al. (2019) found that the composition of marine planktonic bacterial communities across temperature gradients exhibited high heterogeneity in Daya Gulf. However, community assembly and species coexistence of marine bacterial communities remain poorly understood.

Community complexity and stability are intricately tied to species coexistence, because species interactions enhance ecosystem resilience, thereby affecting resistance to environmental stress (Feng et al., 2017). Furthermore, the process of community assembly assumes a pivotal and irreplaceable role in shaping the complexity and ensuring the stability of ecological communities (Chen Z. et al., 2024). Ecosystem complexity is generally characterized by the diversity of species and interconnections within a system, whereas stability pertains to the capacity of the ecosystem to sustain its functional integrity and structural coherence in response to external perturbations (Pettersson, 2023). Numerous studies have shown that different degrees of environmental variation generally promote or inhibit species coexistence and affect ecological complexity and stability (Liu et al., 2021). For example, Naik et al. (2022) found that biotic factors contribute to the overall community stability of marine bacteria. Wang et al. (2024) also demonstrated that prokaryotic plankton in the Yellow Sea and Bohai Bay showed greater network complexity and stability than microeukaryotes, primarily driven by temperature gradients. However, the tipping points for the bacterial community to resist community stress in subtropical Gulfs remain unclear.

Understanding environmental thresholds and their response mechanisms is crucial for identifying fragile tipping points in ecosystems. The environmental threshold refers to the critical point at which environmental factors cause abrupt shifts in community structure and function (Li et al., 2017). Environmental threshold responsiveness could be an important means to study microbial diversity and community stress resistance and to help determine the niche limit (Bardgett and Caruso, 2020). Segmented regression is an effective method for identifying environmental thresholds and is widely used in ecology (Toms and Lesperance, 2003). Cao et al. (2017) identified a pH threshold of 7.74 in plateau lake ecosystems, using segmented regression analysis, and when exceeding the threshold, cyanobacterial community structure was significantly altered and microbial abundance declined. Similarly, Ji et al. (2023) determined thresholds for TN (0.23 ± 0.091 mg/L), DIN (0.21 ± 0.084 mg/L), and NH_4_^+^-N (0.09 ± 0.057 mg/L) in coastal seawater bacterial communities through the same method, and these tipping points corresponded with the bloom dynamics of *Ceratium tripos* and *Skeletonema costatum*. However, the tipping points for marine bacterial communities to environmental stress remain unknown, particularly in subtropical bay ecosystems.

Beibu Gulf is located in the northwest region of the South China Sea and has abundant marine resources and complex microbial community structures (Li et al., 2020; Qin et al., 2024). In recent years, frequent human activities in the Beibu Gulf area have led to temporal and spatial environmental heterogeneity, potentially leading to an imbalance in resource distribution within the area (Lai et al., 2014). Therefore, to investigate the impact of environmental heterogeneity on marine microbial diversity and to identify the tipping points associated with resistance to environmental stress, we collected seawater samples from various layers in the Beibu Gulf to (i)delineate the co-occurrence patterns of bacteria in the Beibu Gulf, (ii)reveal the main factors influencing marine microbial network complexity and stability in the Beibu Gulf, and (iii)explore the environmental inhibition and environmental thresholds of marine microbial community diversity, community complexity, and stability. Overall, this study provides new insights into marine microbial diversity and community stress resistance in the Beibu Gulf and provides an important ecological perspective for the observation of marine ecosystems.

## 2 Materials and methods

### 2.1 Sampling sites and environmental parameters

**Figure 1:**
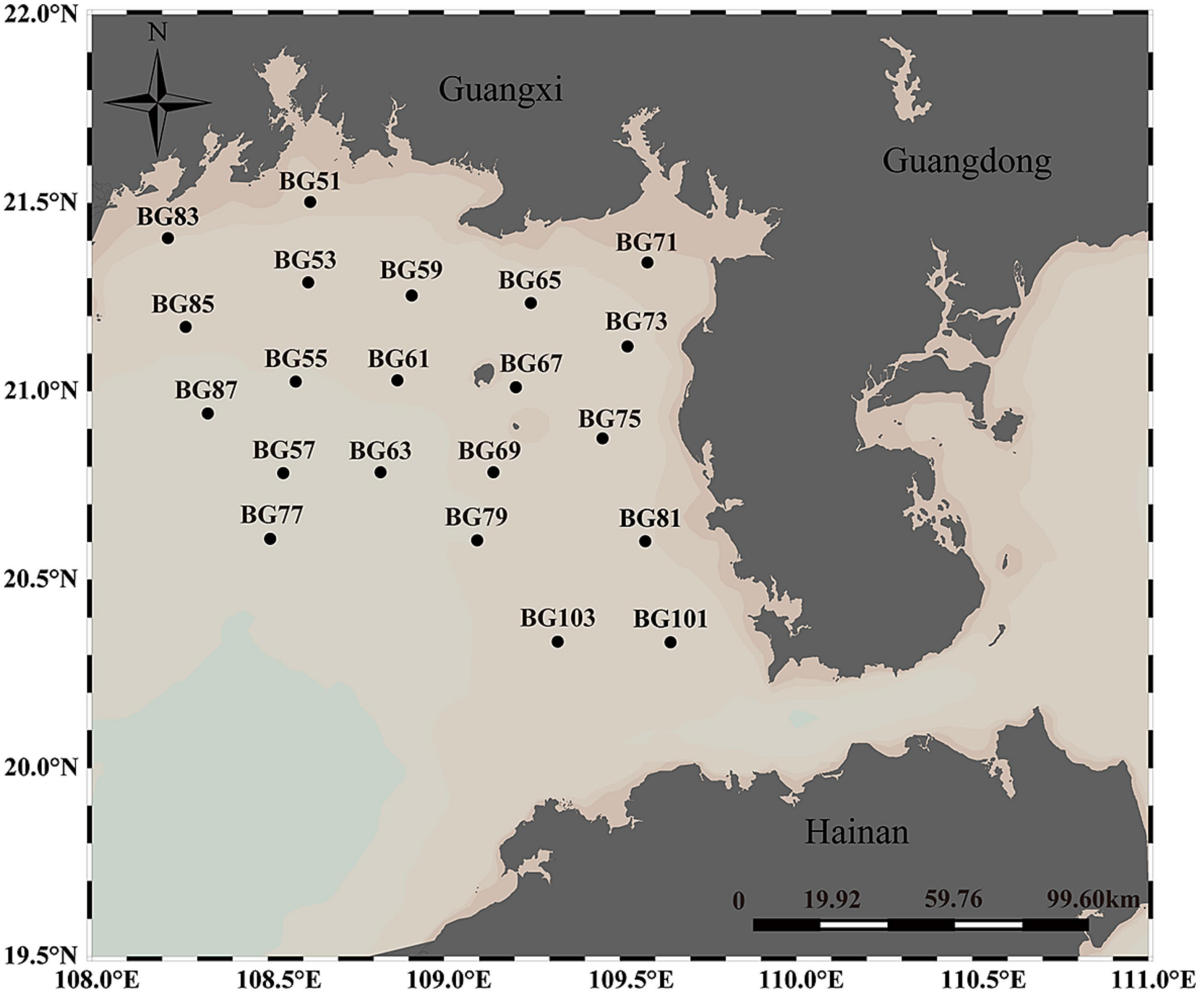
Distribution of 21 sampling sites of the Beibu Gulf in the Northwest of the South China Sea.

In total, 275 samples from 21 sites were collected from seawater at various depths during open cruises of Beibu Gulf on August 10, 2021 (Figure 1). Seawater was sampled from each site, and the samples were divided into three categories: the surface layer (SL, 3 ± 0.5 m), the middle layer (ML, 15 ± 0.5 m to 25 ± 0.5 m), and the bottom layer (BL, 30 ± 0.5 m to 40 ± 0.5 m). The SL, ML, and BL groups comprised 21, 16, and 19 sites, respectively. At each site, five replicate water samples were collected using a SBE 32 Carousel Water Sampler. All seawater samples were stored at 4°C before bacteria isolation and the analysis of environmental factors. For analyzing bacterial communities in seawater, a vacuum pump was employed to sequentially filter 1 liter of seawater from each sample through 3-μm filters (Port Washington, NY, United States) to eliminate debris and larger organisms. The filtered samples were subsequently collected on 0.22-μm polycarbonate membranes (Millipore Corporation, Billerica, MA, United States). Environmental parameters of the samples were assessed using a portable meter (556 MPS; YSI, United States) to measure temperature, salinity, pH, and dissolved oxygen (DO). Additionally, concentrations of phosphate (PO₄^3−^-P), nitrite (NO₂^−^-N), nitrate (NO₃^−^-N), and ammonium (NH₄^+^-N) were determined using a continuous flow analyzer (Seal-AA3, Germany). Chlorophyll a (Chl-*a*) was measured according to established methods (American Public Health Association, 1926). Total organic carbon (TOC) was quantified using a TOC-VCPH analyzer (Shimadzu, Japan), and chemical oxygen demand (COD) was measured using alkaline potassium permanganate (KMnO₄). Dissolved inorganic nitrogen (DIN) was calculated as the sum of NO₂^−^-N, NO₃^−^-N, and NH₄^+^-N, while dissolved inorganic phosphorus (DIP) was represented by PO₄^3−^-P.

### 2.2 DNA extraction and PCR amplification

Genomic DNA extraction from seawater samples was conducted using the DNeasy PowerWater Kit (QIAGEN, United States) in conjunction with 0.22-μm polycarbonate membranes, following the manufacturer’s instructions. The V3 and V4 regions of the 16S rRNA genes were amplified using the primer set 16S-F (5′-AGAGTTTGATCMTGGCTCAG-3′) and 16S-R (5′-TACGGYTACCTTGTTACGACTT-3′) (Cole et al., 2013). The PCR mixture, totaling 20 μL, comprised 2 μL of DNA template, 6 μL of ddH₂O, 10 μL of 2 × Taq PCR Mastermix (TianGen, China), and 2 μL each of the forward and reverse primers. PCR amplification was performed using a Bio-Rad thermocycler (Hercules, CA, United States) with the following protocol: an initial activation step at 94°C for 1 min, followed by 35 cycles of denaturation at 95°C for 30 s, annealing at 56°C for 30 s, and extension at 72°C for 30 s, concluding with a final elongation step at 72°C for 10 min. Ultrapure water served as the negative control to eliminate the potential for false-positive results. The PCR products were validated using 2% agarose gel electrophoresis and visualized under UV light with a gel imaging system.

### 2.3 High-throughput sequencing

A clean library was generated following the standard Illumina library preparation protocols and subsequently sequenced on the Illumina MiSeq platform at Majorbio Co., Ltd. (Shanghai, China). Sequences containing low-quality reads were filtered out using the DADA2 denoising algorithm within the Qiime2 framework (Caporaso et al., 2010). For downstream analyses, amplicon sequence variants (ASVs) derived from the Illumina amplicon dataset were employed without applying arbitrary dissimilarity thresholds (Callahan et al., 2017). Taxonomic assignment of ASVs was conducted by performing a local BLASTN search (with a cutoff E-value of 1e^−10^) against the silva 16S database (Sun et al., 2022). All sequence data have been deposited in GenBank under the BioProject Accession number PRJNA1242707.

### 2.4 Statistical analysis

We calculated the Richness, Shannon, Simpson, and Chao1 (Good, 1953; Kemp and Aller, 2004) indices, and using the Simpson index to represent the alpha diversity. The effect of environmental factors on prokaryotic communities was measured using the Mantel test. Bray–Curtis dissimilarity was calculated to refer to beta diversity. Both of alpha-diversity and beta-diversity were shown by boxplots using “ggplot2” package. Correlations were calculated using Spearman’s rank method. Use R package “ggplot2” for linear regression analysis. As mentioned earlier, null model analysis was performed to classify the community assembly process (Stegen et al., 2013). Based on phylogenetic and taxonomic characteristics, the beta diversity metrics using the β-nearest taxon index (βNTI) and Bray-Curtis-based Raup-Crick, were generated for the evaluation of community assembly. Network analyses of prokaryotes were performed using “Hmisc” package, and visualized using Gephi 0.9.2 software. Spearman heatmaps were fabricated using “pheatmap” package to show the relationship between environmental factors and network properties, particularly complexity and stability. Using a segmented regression analysis, we determined that the relationship followed trend (Gunst and Mason, 2018) and established environmental thresholds.

## 3 Results

### 3.1 Composition and diversity of marine bacteria in Beibu Gulf

**Figure 2:**
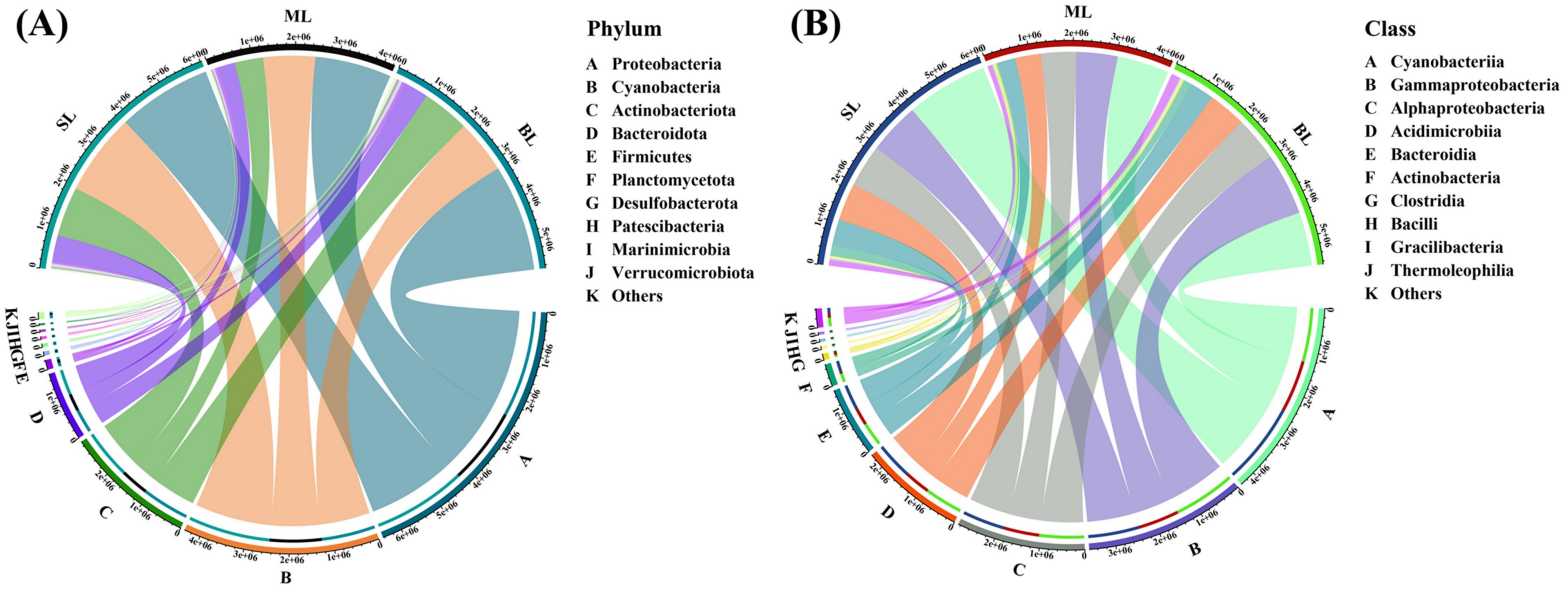
**(A)** Relative abundance of marine bacteria in seawater samples from the Beibu Gulf in phylum level; **(B)** Relative abundance of marine bacteria in seawater samples from the Beibu Gulf in class level. SL: Surface layer water; ML: Middle layer water; BL: Bottom layer water.

In this study, 275 water samples from different depths were collected from Beibu Gulf. Ultimately, 1,456,439 high-quality sequences were obtained. At the phylum level, Proteobacteria (40.38%) was dominant, followed by Cyanobacteria (27.35%), Actinobacteriota (18.24%), and Bacteroidota (9.71%) (Figure 2). Notably, as the sample depth increased, the relative abundance of Proteobacteria gradually increased, whereas that of Cyanobacteria consistently decreased. At the class level, Cyanobacteria (27.35%), Gammaproteobacteria (22.49%), and Alphaproteobacteria (17.89%) were dominant in all three sample groups (Figure 2). Furthermore, both Cyanobacteria and Gammaproteobacteria exhibited a vertical decrease in relative abundance with increasing seawater depth.

**Figure 3:**
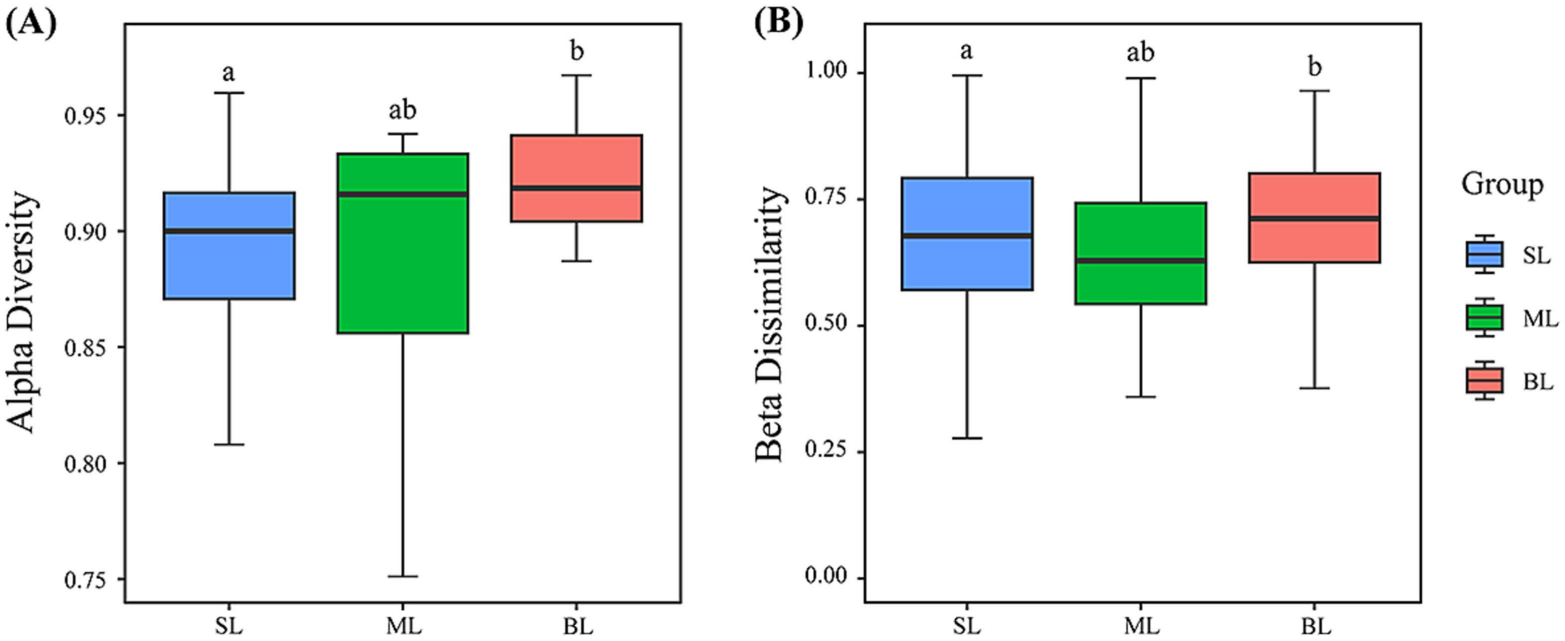
**(A)** Alpha diversity (Simpson index) presented by boxplot; **(B)** Beta dissimilarity (Bray-Curtis distance) presented by boxplot. In the box plots, the upper whisker represents the maximum value, the upper line of the box represents the upper quartile, the center line inside the box represents the median, the lower line of the box represents the lower quartile, and the lower whisker represents the minimum value. Different lowercase letters represent significant differences (*p* < 0.05), and the same lowercase letters indicate no significant differences (*p* < 0.05). SL: Surface layer water; ML: Middle layer water; BL: Bottom layer water.

The Simpson index was used to assess the diversity of marine bacteria. The results revealed that the BL group exhibited the highest alpha diversity, followed by the ML and SL groups (Figure 3A). The ML group showed the lowest levels of beta diversity, followed by the SL group, with the BL group showing the highest levels (Figure 3B).

### 3.2 Assembly process of marine bacteria community in Beibu Gulf

**Figure 4:**
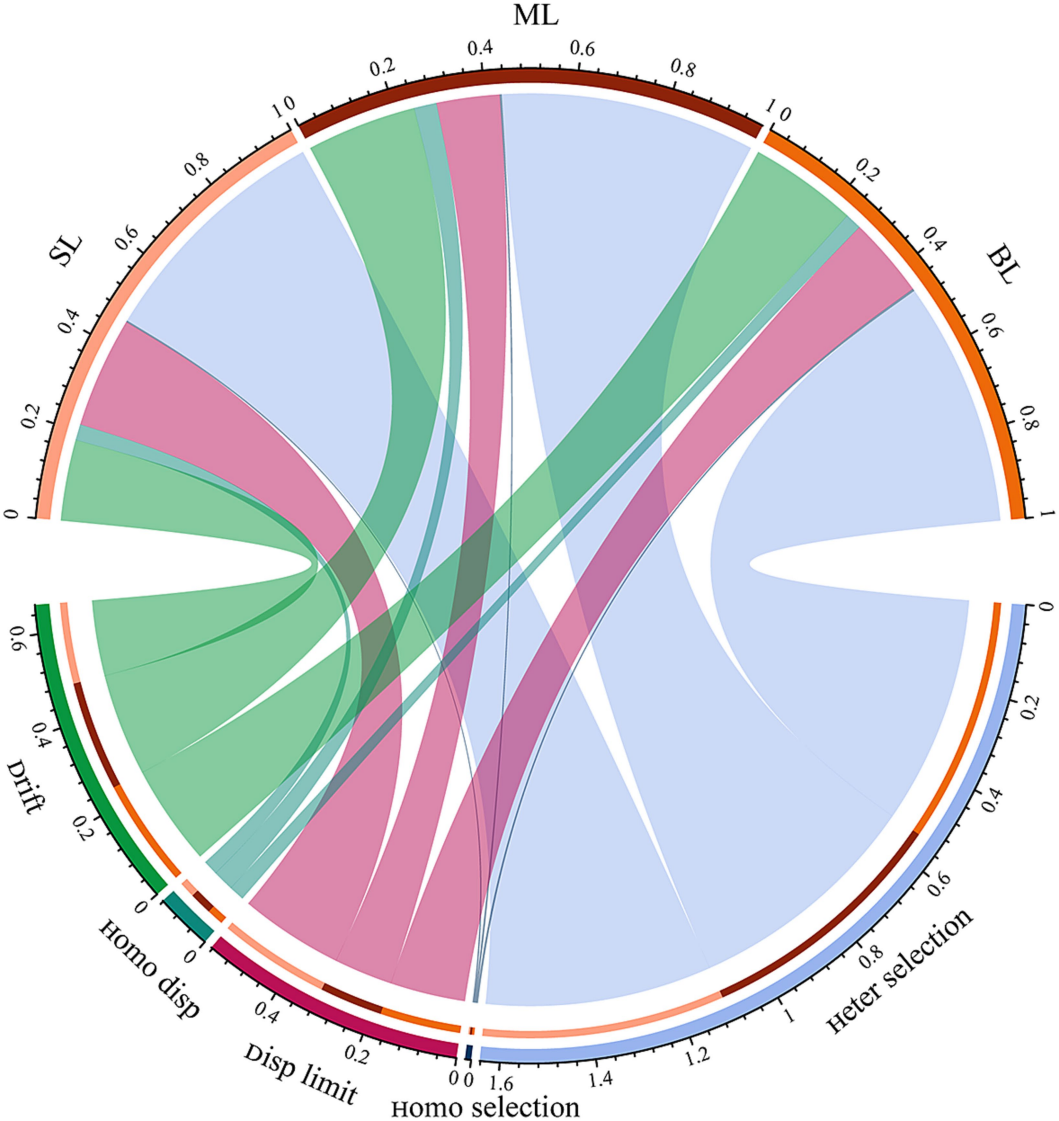
Analysis of the marine bacterial community assembly in different layers. SL: Surface layer water; ML: Middle layer water; BL: Bottom layer water.

NTI was performed to evaluate the relative importance of stochastic and deterministic processes in shaping the assembly of marine bacterial communities in different habitats. |βNTI| ≥ 2 and |βNTI| ≤ 2 represent dominant deterministic processes and stochastic processes in shaping the marine bacteria community, respectively. The proportions of βNTI values between > 2 or < −2 were 54.06, 56.75, and 54.61% for the marine bacteria community in SL, ML, and BL, respectively (Figure 4; Supplementary Table S1). Heterogeneous selection dominated the marine bacterial communities, accounting for 53.71, 56.32, and 54.12%, respectively (Figure 4; Supplementary Table S1). Deterministic rather than stochastic processes dominate the assembly of marine bacterial communities in different habitats. Additionally, ecological drift was second only to heterogeneous selection, accounting for 17.89, 24.31, and 23.90% of the variance. The importance of disp limit was higher in SL samples (24.52%) than in BL samples (17.83%) and ML samples (13.95%) (Figure 4; Supplementary Table S1). In general, deterministic processes contribute more to the dynamics of marine bacterial communities in different habitats than stochastic processes. Spearman’s correlation analyse was performed to explore the key drivers of βNTI. The results showed that DO (*r*^2^ = 0.25, *p* < 0.001) had the greatest impact on βNTI in the SL samples, TOC (*r*^2^ = 0.38, *p* < 0.001) and DO (*r*^2^ = 0.27, *p* < 0.001) had the significant effect on βNTI in the ML samples, whereas DO (*r*^2^ = 0.41, *p* < 0.001) had the strongest effect on alpha diversity in the BL samples (Supplementary Table S2). Collectively, these observations suggest that DO is the key factor for βNTI of marine bacteria across layers.

### 3.3 Network topological features of marine bacteria community in three layers

**Figure 5:**
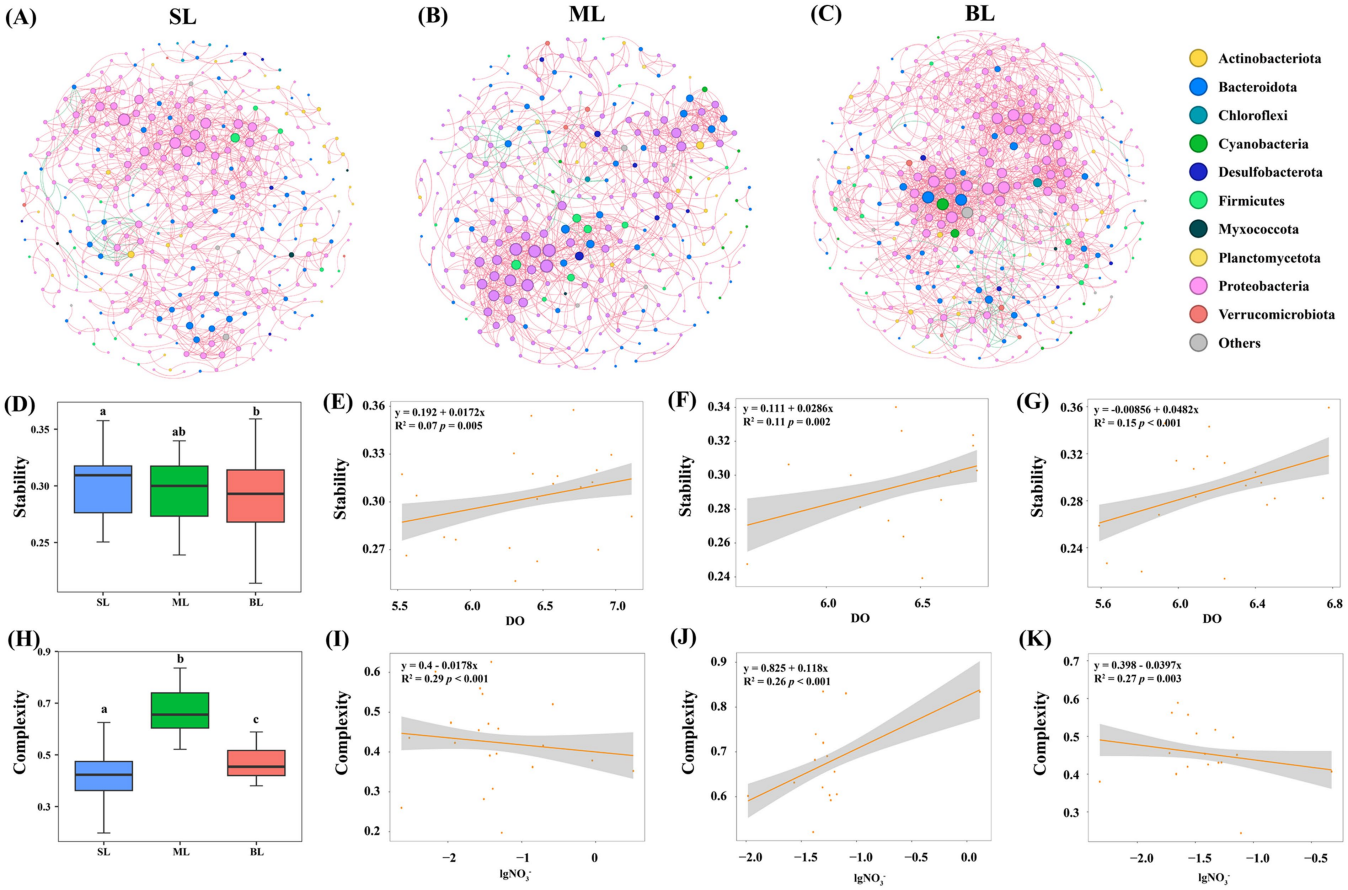
**(A–C)** Co-occurrence network of the three groups. The node colors indicate species from the same module in each network. The lines indicate positive (pink) and negative (green) correlation coefficients. **(A)** SL: Surface layer water; **(B)** ML: Middle layer water; **(C)** BL: Bottom layer water. **(D)** Stability in different layers presented by boxplot. **(E–G)** Linear regression for DO associated with stability in different layers. **(E)** SL: Surface layer water; **(F)** ML: Middle layer water; **(G)** BL: Bottom layer water. **(H)** Complexity in different layers presented by boxplot. **(I–K)** Linear regression for nitrate associated with the complexity in different layers. **(I)** SL: Surface layer water; **(J)** ML: Middle layer water; **(K)** BL: Bottom layer water.

Three co-occurrence networks were constructed for each sample type (SL, ML, and BL) among bacterial OTUs (Figure 5). The BL network had the greatest average degree (8.144) and density (0.023), followed by the ML (average degree = 6.247 and density = 0.021) and SL networks (average degree = 5.363 and density = 0.015) (Supplementary Table S3). Moreover, the SL networks showed a greater number of modules (15) and modularity indices (0.719) than the ML (module = 12, modularity = 0.703) and BL (module = 4, modularity = 0.659) networks (Supplementary Table S3). The ML network had the highest clustering coefficient (0.514), whereas the BL network had the lowest (0.460). Cohesion and robustness are regarded as network complexity and stability, respectively (Supplementary Table S3). The ML network had the highest complexity (0.678), followed by the BL (complexity = 0.470) and SL networks (complexity = 0.424) (Supplementary Table S3). However, the SL network had the highest stability (0.302), followed by the ML (stability = 0.294) and BL (stability = 0.290) networks (Supplementary Table S3).

### 3.4 Driving factors and thresholds of biodiversity, assembly and community stress resistance of marine bacteria

Spearman’s correlation and Mantel test analyses were performed to explore the key drivers of biodiversity, community complexity, and stability of marine bacteria. The results showed that pH (*r*^2^ = −0.51, *p* < 0.001) had the greatest impact on alpha diversity in the SL samples, TN (*r*^2^ = −0.42, *p* < 0.001) and PO_4_^3−^ (*r*^2^ = 0.41, *p* < 0.001) had the significant effect on alpha diversity in the ML samples, whereas temperature (*r*^2^ = −0.37, *p* < 0.001) had the strongest effect on alpha diversity in the BL samples (Supplementary Table S4).

The beta diversity of bacterial communities is determined by various water properties and nutrients. In the SL samples, the beta diversity was mostly influenced by salinity (*r*^2^ = 0.39, *p* < 0.001) and DO (*r*^2^ = 0.44, *p* < 0.001) (Supplementary Table S4). Temperature (*r*^2^ = 0.49, *p* < 0.001) and DO (*r*^2^ = 0.59, *p* < 0.001) had the most significant effects on alpha diversity in ML samples. In the BL samples, temperature (*r* = 0.40, *p* < 0.001), DO (*r*^2^ = 0.30, *p* < 0.001), and TN (*r*^2^ = 0.39, *p* < 0.001) had the greatest effects on beta diversity (Supplementary Table S5). These results indicate that DO is the main driver of marine microbial beta dissimilarities.

**Figure 6:**
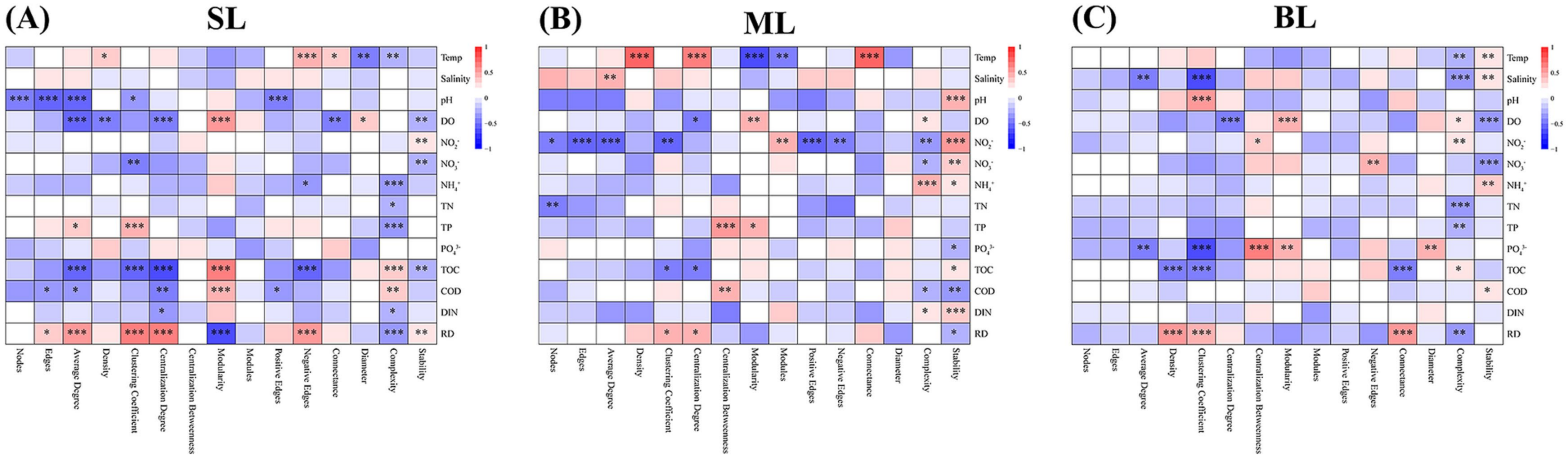
Correlations between stability, complexity, and environmental factors. **(A)** SL: Surface layer water; **(B)** ML: Middle layer water; **(C)** BL: Bottom layer water. Temp: temperature; pH: pH value; DO: Dissolved oxygen; NO_2_^−^: Nitrite-nitrogen; NO_3_^−^: Nitrate-nitrogen; NH_4_^+^: Ammonium-nitrogen; TN: Total nitrogen; PO_4_^3−^: Phosphorus; TOC: Total organic carbon; TIC: Total inorganic carbon; COD: Chemical oxygen demand; DIN: Dissolved inorganic nitrogen. *p < 0.05; **p < 0.01; ***p < 0.001.

The Spearman’s rank method was used to explore the key drivers of community complexity and stability in marine bacteria. In the SL samples, community stability was significantly influenced by DO (*r*^2^ = 0.23, *p* < 0.05), TP (*r*^2^ = −0.39, *p* < 0.001) and RD (*r*^2^ = −0.39, *p* < 0.001), while community complexity was influenced by NO_2_^−^-N (*r*^2^ = 0.29, *p* < 0.01) and NO_3_^−^-N (*r*^2^ = 0.27, *p* < 0.01) (Supplementary Table S4; Figure 6). In the ML samples, DO (*r*^2^ = 0.29, *p* < 0.01) and NH_4_^+^-N (*r*^2^ = 0.44, *p* < 0.001) significantly affected community stability, whereas NO_2_^−^-N (*r*^2^ = 0.53, *p* < 0.001) and NO_3_^−^-N (*r*^2^ = 0.32, *p* < 0.01) had the strongest effects on community complexity (Supplementary Table S4; Figure 6). In the BL samples, community stability was significantly influenced by salinity (*r*^2^ = −0.39, *p* < 0.001) and DO (*r*^2^ = 0.26, *p* < 0.05), while community complexity was mostly influenced by DO (*r*^2^ = −0.39, *p* < 0.001) and NO_3_^−^-N (*r*^2^ = −0.39, *p* < 0.001) (Supplementary Table S4; Figure 6). Collectively, these observations suggest that DO and NO_3_^−^-N are key factors for community stability and complexity of marine bacteria, respectively.

**Figure 7:**
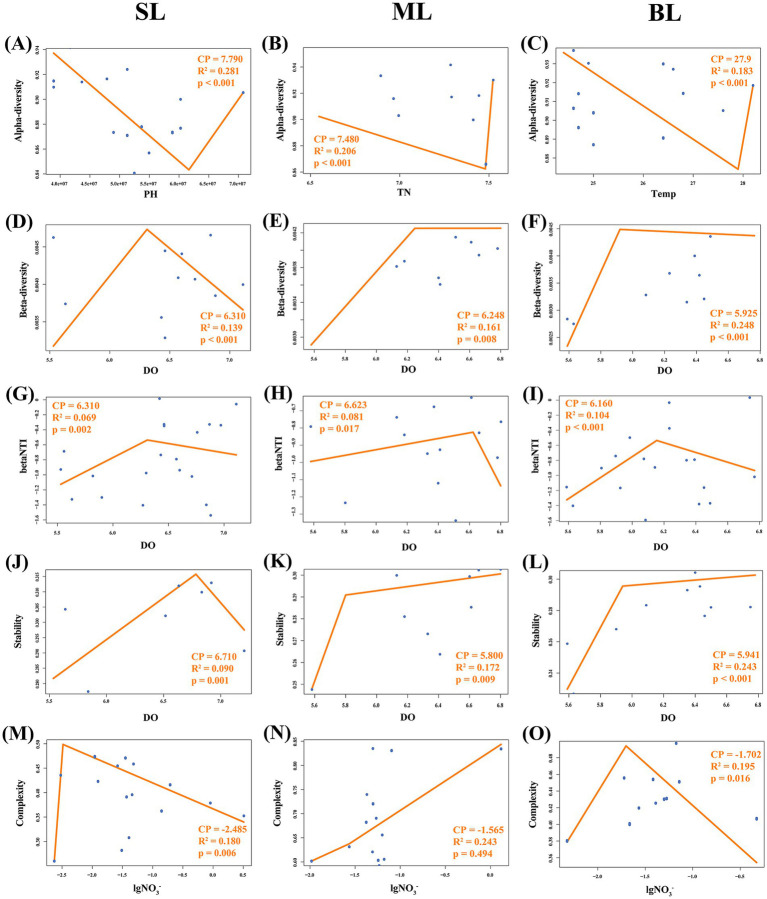
**(A–C)** Segmented regressions for the dominant environmental factors associated with alpha dissimilarity in the different layers. **(A)** SL: Surface layer water; **(B)** ML: Middle layer water; **(C)** BL: Bottom layer water. **(D–F)** Segmented regressions for DO associated with beta dissimilarities in different layers. **(D)** SL: Surface layer water; **(E)** ML: Middle layer water; **(F)** BL: Bottom layer water. **(G–I)** Segmented regressions for DO associated with βNTI in different layers. **(G)** SL: Surface layer water; **(H)** ML: Middle layer water; **(I)** BL: Bottom layer water. **(J–L)** Segmented regressions for DO associated with stability in different layers. **(J)** SL: Surface layer water; **(K)** ML: Middle layer water; **(L)** BL: Bottom layer water. **(M–O)** Segmented regressions for nitrate associated with the complexity in different layers. **(M)** SL: Surface layer water, **(N)** ML: Middle layer water, **(O)** BL: Bottom layer water.

The tipping points of marine bacteria responsive to crucial environmental variables in Beibu Gulf were determined using segmented regression analysis. Segmented linear regression analysis explored the environmental threshold of alpha diversity were pH = 7.79 (*r*^2^ = 0.28, *p* < 0.001), TN = 7.48 mg/L (*r*^2^ = 0.21, *p* < 0.001) and temperature = 27.9°C (*r*^2^ = 0.18, *p* < 0.001) for the SL, ML, and BL groups, respectively (Figures 7A–C). Change points of DO were 6.31 mg/L (*r*^2^ = 0.14, *p* < 0.001), 6.25 mg/L (*r*^2^ = 0.16, *p* = 0.008), 5.93 mg/L (*r*^2^ = 0.25, *p* < 0.001) for the beta-diversity of marine bacteria in SL, ML, and BL, respectively (Figures 7D–F). For the correlation between DO and βNTI, the change points explored at 5.63 mg/L in SL (*r*^2^ = 0.19, *p* = 0.410), 6.57 mg/L in ML (*r*^2^ = 0.41, *p* < 0.001), and 6.24 mg/L in BL (*r*^2^ = 0.45, *p* < 0.001) (Figures 7G–I). Segmented linear regression analysis also showed significant threshold on the correlation between DO and beta stability at 6.71 mg/L (*r*^2^ = 0.09, *p* = 0.001) in SL group, while insignificant change points were 5.80 mg/L (*r*^2^ = 0.17, *p* = 0.009) and 5.94 mg/L (*r*^2^ = 0.24, *p* < 0.001) in ML and BL, respectively (Figures 7J–L). For NO_3_^−^-N correlated with community complexity, segmented analysis explored the change points at 0.003 in SL (*r*^2^ = 0.18, *p* = 0.006), 0.027 in ML (*r*^2^ = 0.24, *p* = 0.494), and 0.020 in BL (*r*^2^ = 0.20, *p* = 0.016) (Figures 7M–O).

## 4 Discussion

### 4.1 Proteobacteria dominated the Beibu Gulf and deterministic processes governed bacterial community assembly across habitats

Proteobacteria, cyanobacteria, actinobacteria, and Bacteroidetes were the predominant classes in seawater samples from the Beibu Gulf area. Recently, several studies have been conducted on marine bacteria, particularly in subtropical gulfs affected by anthropogenic activities (Yang et al., 2024). For example, Peng et al. (2024) found that Proteobacteria was the dominant bacterial phylum in the Beibu Gulf in both wet and dry seasons, followed by Bacteroidetes and Actinobacteria. Li et al. (2020) reported that Alphaproteobacteria and Gammaproteobacteria are the most abundant classes in Beibu Gulf seawater samples across a eutrophication gradient. Our results indicated that as the seawater samples were taken from the surface to the bottom, the relative abundance of Proteobacteria gradually increased, while the relative abundance of Cyanobacteria continued to decrease. Previous studies have demonstrated that the high photosynthetic activity of cyanobacteria enables them to grow and reproduce rapidly in shallow seawater (Bullerjahn and Post, 2014). Therefore, we inferred that the decline in cyanobacterial abundance in the bottom layer could be attributed to reduced light intensity and dissolved oxygen levels (Wang et al., 2021). Furthermore, the increased dominance of Proteobacteria in the bottom layer may be due to their metabolic versatility, which enables adaptation to reduced dissolved oxygen levels and organic matter deposition (Capo et al., 2020). Overall, the distribution of the dominant species in Beibu Gulf exhibited spatial heterogeneity.

Our findings suggest that biodiversity is significant in different habitats. Although BL samples are remote from direct nutrient inputs, they can become eutrophic through processes such as vertical mixing and settlement effects under certain circumstances. Reissmann et al. (2009) also showed eutrophication from vertical mixing in the Baltic Sea, because sediment organic matter decomposition provides nutrients to bottom-water microorganisms. The accumulation of these nutrients leads to an increase in microbial diversity in bottom layer waters. The research of Jørgensen (2000) has shown that sediments and dissolved organic matter in bottom waters provide abundant nutrition, supporting the growth of a wider variety of microorganisms, particularly anaerobic and facultative anaerobic bacteria. This finding corroborates previous evidence that the relative abundance of Proteobacteria in the BL samples increased. Overall, our findings highlight the influence of environmental perturbations on the distribution and diversity of marine bacterial communities in the Beibu Gulf.

Community assembly was intricately intertwined with species composition, as they collectively offer insights into the processes underpinning the emergence and persistence of biodiversity within ecological systems (Bonte et al., 2024). In this study, we employed a null-modeling-based framework to investigate the assembly process of marine bacterial communities. Our findings revealed that deterministic processes play a pivotal role in driving the turnover of the marine bacterial community across all three layers, with heterogeneous selection regarded as the dominant factor. Heterogeneous selection is a deterministic concept pertaining to distinct environmental selective forces that can propel a community toward increased dissimilarity (Dini-Andreote et al., 2015). Qin et al. (2024) found that deterministic rather than stochastic processes dominated the assembly of marine *Vibrio* communities in different habitats in Beibu Gulf. Yang et al. (2024) also found that deterministic assembly processes predominantly shape microbial communities in the subtropical estuaries of Beibu Gulf. Yuan et al. (2021) and Yan et al. (2024) found that strong selective pressures shape the heterogeneous selection of marine bacterial communities. Our research showed that βNTI was significantly correlated with most water properties, especially DO. Previous studies have demonstrated a strong correlation between water properties and marine bacterial communities (Chun-Yi et al., 2012). Therefore, we propose that deterministic processes play a dominant role in the microbial community assembly through heterogeneous selection, and DO regulate the community assembly in the Beibu Gulf region.

Stochastic processes also play a role in driving the structure of microbial communities (Yuan et al., 2019). Notably, our findings show that stochastic processes, particularly ecological drift, also play an important role in the bacterial communities in the middle and bottom layers of seawater, accounting for 45.94, 43.24, and 45.39% in the three layers, respectively. This effect is due to several factors. First, ecological drift may be more pronounced owing to reduced microbial dispersal, dominance of physical mixing, and a more homogeneous distribution of environmental factors that limit selective pressures in deeper water layers (Zhao et al., 2022). Secondly, the connectivity and mobility of seawater are positively correlated with ecological drift (Dini-Andreote et al., 2015; Zeng et al., 2019). Shi et al. (2018) indicated that deterministic processes are more likely to occur at larger scales, whereas stochastic processes tend to occur at local scales. Therefore, the microbial community structure in Beibu Gulf enhances the influence of stochastic processes, particularly ecological drift, by vertical mixing. Conversely, the relatively low homogenizing dispersal and homogeneous selection observed across all layers indicate the environmental heterogeneity among communities (Soininen and Graco-Roza, 2024). This implies that the bacterial communities in the Beibu Gulf are highly susceptible to the environmental disturbances. Overall, these findings highlight the dominant role of deterministic processes in shaping the marine bacterial community assembly across different water layers and underscore the contribution of stochastic processes in regulating the microbial community structure in the Beibu Gulf.

### 4.2 Nutrient and water properties affecting the diversity and community of marine bacteria

The cohesion index was regarded as an index to reflect community complexity, reflecting strong interactions between microorganisms within a network. In the present study, the ML network exhibited the highest clustering coefficient (0.514) among the various components of the bacterial community. These results suggest that the bacterial community in the middle layer (ML) experienced the strongest interactions and highest degree of community complexity. These results imply that the middle layer provides an environment conducive to enhanced niche sharing and more frequent interspecific interactions among marine bacteria than other layers (Capo et al., 2020). This may be because the middle layer was less affected by these drastic changes, providing more stable living conditions for the microorganisms, which, in turn, facilitated the formation of a more complex community structure. The surface layer is significantly affected by sunlight, temperature fluctuations, and ocean surface disturbances, whereas the bottom layer is influenced by low-oxygen conditions and accumulation of organic matter (Hu et al., 2021). In contrast, ML had a stable habitat, which was also supported by the fact that the ML has the highest proportion of positive edges (97.55%). Therefore, the middle layer exhibited higher community complexity.

Robustness, a measure of network stability, was found to be significantly higher in the SL network (0.302) than in the other layers, suggesting that the bacterial community in the SL has greater community stability. This may be due to the stable oxygen supply and high primary productivity in the surface layer, which could result in the highest stability of the surface microbial community (Terrence et al., 2005; Tengfei et al., 2021). All three networks were dominated by positive correlations, highlighting the crucial role of mutualistic symbiosis and cooperation in sustaining ecosystem functions. Symbiotic relationships are essential for maintaining the stability of microbial community (Liu et al., 2022). These results suggest that the SL community showed stronger species interactions, resistance, and resilience, which enhanced the adaptability of bacteria to environmental pressures.

Understanding the relationship between environmental variables and microbial community structure is an important goal in microbial ecology research (Shen et al., 2015). Previous studies have demonstrated a close association between marine bacterioplankton and environmental factors, with these communities responding rapidly to changes in variables such as temperature, nitrogen, and dissolved oxygen (DO) in oceanic environments (Sampaio et al., 2022; Zhao et al., 2024). This study identified pH, TN, and temperature as the primary factors influencing alpha diversity in the SL, ML, and BL groups. Additionally, DO was found to have a substantial impact on beta diversity across all three habitats, whereas temperature was significantly correlated with beta diversity in BL samples. These findings align with those of previous research on the relationship between environmental variables and microbial community structure. Peng et al. (2024) reported that temperature and DO are critical environmental factors that affect bacterial communities in Beibu Gulf. Temperature, which is a driver of microbial diversity, was also noted by Gusareva et al. (2019) in the Global Ocean System Survey. Jing et al. (2019) found a significant positive correlation between the coastal bacterioplankton community in the Yellow Sea and both temperature and COD, suggesting that COD levels may influence bacterioplankton community structure and diversity. Overall, this study demonstrated that nutrient and water properties significantly shape the alpha and beta diversities of microbial communities across distinct habitats in Beibu Gulf.

Previous studies have consistently confirmed that environmental factors have a crucial influence on the structure of marine bacterial communities (Letscher et al., 2015; Lindh et al., 2016). Our research indicated that DO and NO3-emerges as the crucial determinant of the stability and complexity of marine bacterial communities, respectively. Dissolved oxygen (DO) is a crucial environmental factor that affects the survival and metabolism of marine microorganisms. Higher DO levels help to sustain aerobic microorganisms, thereby maintaining a relatively stable bacterial community structure (Ye and Shi, 2020). In contrast, a decrease in DO may lead to the proliferation of anaerobic microorganisms, causing dynamic shifts in the microbial community and reducing its stability (Jørgensen, 2000). Moreover, DO is closely linked to organic matter decomposition and redox conditions, which likely influence the stability of bacterial communities (Chen Y. et al., 2024). DO plays a dominant role in community stability, whereas community complexity was significantly influenced by nitrate levels. As an essential inorganic nitrogen source, nitrate also plays a key role in driving the nitrogen cycle in marine ecosystems by affecting bacterial metabolic pathways and community composition (Hong, 2013). Higher nitrate levels may support the coexistence of diverse microbial groups with different metabolic capabilities, such as nitrifying bacteria, denitrifying bacteria, and facultative anaerobes (Yi et al., 2018), thereby increasing community diversity and interactions and enhancing the complexity of the marine microbial community. Overall, DO and nitrate plays dominant roles in the stability and complexity of the marine bacterial community, respectively.

### 4.3 Dissolved oxygen and nitrate gradient regulate community stress resistance of marine bacterial communities in Beibu Gulf

Environmental changes regulate the community diversity and stress resistance. Our research indicates that pH, TN, and temperature are the main driving factors controlling the alpha diversity of marine bacterial communities in SL, ML, and BL. According to the segmented regression analysis, we observed that alpha diversity within the three layers exhibited a declining trend before the tipping points but exhibited a rebound post-threshold. This may be caused by pronounced environmental filtering under lower environmental conditions (Song et al., 2019). Therefore, we consider pH = 7.79, TN = 7.48 mg/L, and temperature = 27.9°C to be the respective thresholds of maximum environmental stress for the SL, ML and BL groups. Beyond the tipping point, strengthened interspecies interactions may drive a resurgence in biodiversity (Gorter et al., 2020). Wan et al. (2021) also found that 20.9 to 25.2°C as the bacterioplankton community threshold, but lower than our research. This difference may be caused by the unique climate and temperature variations in subtropical gulfs compared to temperate regions.

DO mainly drive the beta dissimilarity in marine bacterial communities in Beibu Gulf. The results of the segmented regression analysis show that DO exhibits a promoting effect on beta diversity across three layers. Beman and Carolan (2013) also found that DO is positively correlated with the beta diversity of bacterial communities. An increase in DO may lead to enhanced niche differentiation and metabolic pathway diversity, ultimately promoting an increase in beta diversity. However, according to the results of the segmented regression analysis, the promoting effect of DO on beta diversity across all depth layers significantly decreased after the tipping points of 5.93 mg/L-6.31 mg/L. This might be because DO reaches a certain threshold and the bacterial community has already adapted to higher oxygen levels. At this tipping point, any further increase in DO had a diminished effect on community promotion, as the bacteria had already reached their optimal growth state under the current DO conditions. Therefore, we can consider DO concentrations of 6.31 mg/L, 6.25 mg/L and 5.93 mg/L as the optimal growth thresholds for the bacterial communities in the SL, ML and BL, respectively. Shen et al. (2023) also found that most aerobic denitrifiers have an optimal growth state when the DO is 3–5 mg/L. In this study, the DO threshold was higher. This may have been caused by the higher dissolved oxygen background in Beibu Gulf (Zheng et al., 2023), where bacterial communities may have evolved a stronger adaptation to higher oxygen conditions.

βNTI reflects the phylogenetic structural differences in communities and serves as an important ecological indicator for microbial community assembly mechanisms (Xun et al., 2019; Gundersen and Vadstein, 2024). As the key influencing factor of βNTI (Supplementary Table S2), DO concentration exhibits a biphasic effect on community assembly. According to the results of the segmented regression analysis, DO showed significant tipping points in ML and BL. Before the tipping point, community assembly transitions from strong heterogeneous selection to stochastic processes. However, heterogeneous selection becomes strengthened again after the tipping point. This might caused by the functional redundancy of microbial community. When environmental stress in a moderate level, functionally redundant microbial taxa would maintain community structure through compensatory regulation (Louca et al., 2018; Chen et al., 2020), thereby reducing phylogenetic dissimilarity and exhibiting a higher degree of stochasticity (Biggs et al., 2020). Therefore, our study suggests that tipping points at 6.57 mg/L and 6.24 mg/L were phylogenetic stress thresholds in BL in ML.

Similarly, as a main driving factor controlling the stability of marine bacterial communities, DO also show the tipping points across three layers. This indicates that when the DO reached the response threshold for stability, the bacterial community exhibited the strongest species interactions, resistance, and resilience. This may be due to the fact that, prior to reaching the tipping point, increasing DO fosters the growth and metabolic activity of aerobic microorganisms, thereby enhancing community stability (Dvergedal et al., 2020). After reaching the tipping point of 5.94 mg/L-6.71 mg/L, according to the niche saturation effect, oxygen is no longer the main limiting factor and will not significantly alter the microbial niches (Pincheira-Donoso et al., 2018). The LS group had the highest threshold, indicating that marine bacterial communities exhibit greater adaptability to habitats with higher DO contents, contributing to the maintenance of ecosystem stability (Zhu et al., 2023).

Our research also indicated that NO3-serves as a main driving factor controlling community complexity. According to the segmented regression analysis, significant tipping points of NO3-responding community complexity also appeared in the SL and BL samples at 0.003 mg/L and 0.020 mg/L, respectively. This result suggests that the bacterial communities in these two groups had the strongest interactions when they reached their tipping points (Karimi et al., 2017). The absence of a community complexity threshold in the middle layer might be due to an environmental buffering effect (Cai et al., 2017). ML is generally more stable than SL and BL and experiences less disturbance from external environmental changes. As a result, it might cause the absence of community complexity threshold within the middle layer in the regression analysis. In summary, this study demonstrates that bacterial community diversity, community assembly, and environmental resistance are concurrently driven by multiple environmental factors, such as pH, TN, temperature, DO, NO_3_^−^, etc. Even when a single variable exhibits a significant effect, its explanatory power with respect to the response variable is limited, resulting in a relatively low *R*^2^ value. Consequently, despite the low *R*^2^ value obtained from piecewise regression analysis, the results remain statistically significant given the significant *p* values (*p* < 0.05). Overall, the diversity, community stability, and complexity of the marine bacterial communities in Beibu Gulf are primarily regulated by dissolved oxygen and nitrate. The bacterial communities in different water layers exhibited distinct environmental adaptations and ecological response patterns.

## 5 Conclusion

In the present study, the spatial distribution of marine bacteria in different habitats was determined using high-throughput sequencing. Proteobacteria were the most abundant phyla in the seawater of Beibu Gulf. Alpha diversity was higher in the bottom layer, beta diversity was higher in the middle layer, and the water properties were important for the diversity of marine bacteria. Deterministic processes dominate marine bacterial community assembly. DO was the main factor affecting bacterial dissimilarity and community stability, whereas complexity was mainly influenced by NO_3_^−^. Thresholds of pH = 7.79, TN = 7.48 mg/L, and temperature = 27.9°C marked maximum environmental stress for SL, ML and BL communities, while DO concentrations of 6.31 mg/L, 6.25 mg/L and 5.93 mg/L were thresholds for beta diversity and 6.57 mg/L and 6.24 mg/L were thresholds for βNTI in ML and BL. DO concentrations of 6.71 mg/L, 5.80 mg/L and 5.94 mg/L were thresholds for community stability corresponding to peak community interactions. Nitrate-responsive complexity thresholds emerged at 0.003 mg/L in the SL and 0.020 mg/L in the BL but were absent in the middle layer. Overall, these findings constitute a scaffold upon which to better understand the bacterial community structure and community stress resistance responses to changes in environmental factors.

## Data Availability

The original contributions presented in the study are publicly available. This data can be found here: https://www.ncbi.nlm.nih.gov/, accession number: PRJNA1242707.
