# Supplementary material for: Dissolved oxygen and nitrates gradient influence marine microbial complexity and stability in Beibu Gulf
Source: Front Microbiol. 2025 Jun 25;16:1622150. doi: 10.3389/fmicb.2025.1622150 (PMC12237889; doi:10.3389/fmicb.2025.1622150)
Supplement: Supplementary file 1 [file Data_Sheet_1.docx]

**Supplementary Information (R code)**

# Dissolved oxygen and nitrates gradient influence marine microbial complexity and stability in Beibu Gulf

**Qing He^1,a^, Qingxiang Chen^1,a^, Xinyi Qin^2^, Shengyao Zhou^3^, Rajapakshalage Thashikala Nethmini^1^, Gonglingxia Jiang^1^, Qinghua Hou^1^, Xiaolei Li^1^, Laizhen Huang^1^, Ke Dong^4^, Lingling Xie^1^, Nan Li^1*^**

^1^Key Laboratory of Climate, Resources and Environment in Continental Shelf Sea and Deep Sea of Department of Education of Guangdong Province, Department of Oceanography, Key Laboratory for Coastal Ocean Variation and Disaster Prediction, College of Ocean and Meteorology, Guangdong Ocean University, Zhanjiang 524088, China

^2^Key Laboratory of Environment Change and Resources Use in Beibu Gulf, Ministry of Education (Nanning Normal University), Nanning, Guangxi, 530001, China

^3^College of Environmental Science and Engineering, Guilin University of Technology, Guilin, China

^4^Department of Biological Sciences, Kyonggi University, 154-42, Gwanggyosan-ro, Yeongtong-gu, Suwon-si, Gyeonggi-do 16227, South Korea; Republic of Korea

**Correspondence:**

*Corresponding Author: Nan Li

nli0417@163.com

^a^Qing He and Qingxiang Chen contributed equally to this paper and are co-first authors.

**Diversity analysis**

library(vegan)

goods <-

function(com){

no.seqs <- rowSums(com)

sing <- com==1

no.sing <- apply(sing, 1, sum)

goods <- 100*(1-no.sing/no.seqs)

goods.sum <- cbind(no.sing, no.seqs, goods)

goods.sum <- as.data.frame(goods.sum)

return(goods.sum)

}

table1<- read.table(file=file.choose(),sep=",",header=T,row.names=1)

otu=table1

otu=t(otu)

Shannon=diversity(otu,"shannon")

Simpson=diversity(otu,"simpson")

insimp=diversity(otu,"inv")

ss=specnumber(otu)

est=estimateR(otu)

est=t(est)

est1=est[,1:4]

coverage1=goods(otu)

Coverage=coverage1[,c("goods")]

alph=data.frame(Shannon,Simpson,est1,Coverage,check.names = T)

write.csv(alph, file="BBG16S_alph_acID.csv")

**Correlation analysis**

library(psych)

library(xlsx)

x<- read.table(file=file.choose(),sep=",",header=T,row.names=1)

y <- psych::corr.test(x,method = "spearman")

r<-y$r#提取r

p<-y$p#提取p

sig_label<-function(jp){ifelse(jp<0.001,"***",ifelse(jp<0.01,"**",ifelse(jp<0.05,"*","")))}

sig_matrix<-sig_label(p)

r=round(r,2)

rp=paste(r,sig_matrix)

rp1=matrix(rp,nrow=21, byrow=FALSE)

rownames(rp1)<-rownames(r)

colnames(rp1)<-colnames(r)

#write.csv(sig_matrix,file="p.csv")

#write.csv(r,file="r.csv")

write.xlsx(rp1,file="FAL_c_cor.xlsx",sheetName="Sheet1",row.names=T,col.names=T)

**Assembly analysis**

library(iCAMP)

library(picante)

comm1<-read.table('BBG16S-OTU.csv',header=TRUE,sep=",",row.names = 1)comm<-t(comm1)

tree<-read.tree(file="tree.nwk")

nworker=4

pd.big=pdist.big(tree = tree, nworker = nworker)

rand.time=1000

bNTI=bNTI.big(comm=comm, pd.desc=pd.big$pd.file,

pd.spname=pd.big$tip.label,pd.wd=pd.big$pd.wd,

spname.check=TRUE, nworker=nworker, memo.size.GB=5,

weighted=TRUE, exclude.consp=FALSE,rand=rand.time,

output.dtail=FALSE, RC=FALSE, trace=TRUE)

write.csv(bNTI,file="bNTI_iCAMP-BL.csv")

RC=RC.pc(comm=comm, rand = rand.time,

nworker = nworker, weighted = TRUE,

sig.index="RC")

write.csv(RC,file="RC.bray_iCAMP-BL.csv")

**Network analysis**

library(Hmisc)

library(igraph)

OTU <- read.delim('BBG16S-OTU-BL.csv', row.name = 1, check.names = FALSE,sep = ',')

OTU1 <- OTU

OTU1[OTU1>0] <- 1

OTU <- OTU[which(rowSums(OTU1) >= 10), ]

OTU_corr <- rcorr(t(OTU), type = 'spearman')

r <- OTU_corr$r

r[abs(r) < 0.7] <- 0

write.csv(data.frame(r), 'OTU_corr.r-BL.csv')

p <- OTU_corr$P

p[p>=0.01] <- -1

p[p<0.01 & p>=0] <- 1

p[p==-1] <- 0

z <- r * p

diag(z) <- 0

head(z)[1:6,1:6]

write.csv(data.frame(z), 'OTU_corr.matrix-BL.csv')

OTU.net <- graph.adjacency(z, weighted = TRUE, mode = 'undirected')

OTU.net

OTU.net <- simplify(OTU.net)

OTU.net <- delete.vertices(OTU.net, names(degree(OTU.net)[degree(OTU.net) == 0]))

E(OTU.net)$correlation <- E(OTU.net)$weight

E(OTU.net)$weight <- abs(E(OTU.net)$weight)

E(OTU.net)$correlation

E(OTU.net)$weight

tax1 <- read.delim('taxa.anno.csv', row.name = 1, check.names = FALSE, stringsAsFactors = FALSE,sep = ',')

tax=tax1[match(row.names(OTU),row.names(tax1)),]

tax <- tax[as.character(V(OTU.net)$name), ]

V(OTU.net)$phylum <- tax$phylum

V(OTU.net)$class <- tax$class

V(OTU.net)$order <- tax$order

V(OTU.net)$family <- tax$family

V(OTU.net)$genus <- tax$genus

V(OTU.net)$species <- tax$species

V(OTU.net)$acc.num <- tax$acc.num

OTU.net

plot(OTU.net)

adj_matrix <- as.matrix(get.adjacency(OTU.net, attr = 'correlation'))

write.csv(data.frame(adj_matrix), 'network.adj_matrix-BL.csv')

edge <- data.frame(as_edgelist(OTU.net))

edge_list <- data.frame(

source = edge[[1]],

target = edge[[2]],

weight = E(OTU.net)$weight,

correlation = E(OTU.net)$correlation

)

head(edge_list)

edge_list[which(edge_list$correlation > 0), "Egtype"] <- "1"

edge_list[which(edge_list$correlation < 0), "Egtype"] <- "-1"

write.csv(edge_list, row.names = FALSE,'BL.Edge_list.csv')

node_list <- data.frame(

label = names(V(OTU.net)),

phylum = V(OTU.net)$phylum,

class = V(OTU.net)$class,

order = V(OTU.net)$order,

family = V(OTU.net)$family,

genus = V(OTU.net)$genus,

species = V(OTU.net)$species,

acc.num = V(OTU.net)$acc.num

)

head(node_list)

tax.type1 <- read.table(file="OTU.Type.csv",sep=",",header=T,row.names=1)

tax.type <- tax.type1[as.character(node_list$label),]

node_list <- cbind(node_list$label,node_list,tax.type)

colnames(node_list)[1] <- "ID"

write.table(node_list,row.names = FALSE, 'all.Node_list.csv')

write.graph(OTU.net, 'all.network.graphml', format = 'graphml')

write.graph(OTU.net, 'all.network.gml', format = 'gml')

**Segment regression analysis**

library(SiZer)

Arkansas<- read.table(file="BL-B-DO.csv",sep=",",header=T,row.names=1,check.names=F)

library(segmented)

fit_lm <- lm(beta~DO, data = Arkansas)

summary(fit_lm)

lm_seg1 <- segmented(fit_lm, seg.Z = ~DO, npsi = 1)

summary(lm_seg1)

plot(lm_seg1, xlab = 'DO', ylab = 'beta')

points(beta~DO, data = Arkansas)
