# Supplementary material for: Dissolved oxygen and nitrates gradient influence marine microbial complexity and stability in Beibu Gulf
Source: Front Microbiol. 2025 Jun 25;16:1622150. doi: 10.3389/fmicb.2025.1622150 (PMC12237889; doi:10.3389/fmicb.2025.1622150)
Supplement: Supplementary file 2 [file Table_1.docx]

**Supplementary Information (Table S1-S6)**

# Dissolved oxygen and nitrates gradient influence marine microbial complexity and stability in Beibu Gulf

**Qing He^1,a^, Qingxiang Chen^1,a^, Xinyi Qin^2^, Shengyao Zhou^3^, Rajapakshalage Thashikala Nethmini^1^, Gonglingxia Jiang^1^, Qinghua Hou^1^, Xiaolei Li^1^, Laizhen Huang^1^, Ke Dong^4^, Lingling Xie^1^, Nan Li^1*^**

^1^Key Laboratory of Climate, Resources and Environment in Continental Shelf Sea and Deep Sea of Department of Education of Guangdong Province, Department of Oceanography, Key Laboratory for Coastal Ocean Variation and Disaster Prediction, College of Ocean and Meteorology, Guangdong Ocean University, Zhanjiang 524088, China

^2^Key Laboratory of Environment Change and Resources Use in Beibu Gulf, Ministry of Education (Nanning Normal University), Nanning, Guangxi, 530001, China

^3^College of Environmental Science and Engineering, Guilin University of Technology, Guilin, China

^4^Department of Biological Sciences, Kyonggi University, 154-42, Gwanggyosan-ro, Yeongtong-gu, Suwon-si, Gyeonggi-do 16227, South Korea; Republic of Korea

**Correspondence:**

*Corresponding Author: Nan Li

nli0417@163.com

^a^Qing He and Qingxiang Chen contributed equally to this paper and are co-first authors.

**Table S1** Assembly analysis results of marine bacteria community in different layers.

|  | SL | ML | BL |
| --- | --- | --- | --- |
| Heter selection | 53.71% | 56.32% | 54.12% |
| Homo selection | 0.35% | 0.43% | 0.49% |
| Disp limit | 24.52% | 13.95% | 17.83% |
| Homo disp | 3.53% | 4.99% | 3.65% |
| Drift | 17.89% | 24.31% | 23.91% |

SL: surface layer water; ML: Middle layer water; BL: Bottom layer water.

**Table S2** Spearman tests for the correlations between βNTI and the environmental factors.

|  | SL | ML | BL |
| --- | --- | --- | --- |
| Temp | 0.21 *** | 0.09 *** | 0.17 *** |
| Salinity | -0.11 *** | 0.19 *** | 0.06 *** |
| pH | -0.22 ** | 0.17 *** | 0.18 *** |
| DO | 0.25 *** | 0.27 *** | 0.41 *** |
| NO_2_^-^ | -0.09 *** | 0.25 *** | 0.18 *** |
| NO_3_^-^ | -0.01 | 0.22 *** | 0.07 *** |
| NH_4_^+^ | 0.21 *** | 0.26 *** | 0.2 *** |
| TN | 0.09 *** | 0.32 *** | 0.28 *** |
| TP | 0.19 *** | 0.12 *** | 0.17 *** |
| PO_4_^3-^ | 0.06 *** | 0.19 *** | 0.25 *** |
| TOC | 0.03 * | 0.38 *** | 0.03 * |
| COD | 0.05 *** | 0.28 *** | 0.31 *** |
| Chl-*a* | 0.04 ** | 0.34 *** | 0.23 *** |

SL: surface layer water; ML: Middle layer water; BL: Bottom layer water.

**Table S3** Topological parameters of the bacterial co-occurrence networks of the three layers.

| Metric | SL | ML | BL |
| --- | --- | --- | --- |
| Nodes | 357 | 300 | 362 |
| Edges | 960 | 937 | 1474 |
| Average degree | 5.363 | 6.247 | 8.144 |
| Diameter | 17 | 15 | 14 |
| Density | 0.015 | 0.021 | 0.023 |
| Clustering coefficient | 0.484 | 0.514 | 0.46 |
| Modules | 15 | 12 | 4 |
| Modularity | 0.719 | 0.703 | 0.659 |
| Positive edges | 932 | 914 | 1429 |
| Negative edges | 28 | 23 | 45 |
| Complexity | 0.424 | 0.678 | 0.470 |
| Stability | 0.302 | 0.294 | 0.290 |

SL: surface layer water; ML: Middle layer water; BL: Bottom layer water.

**Table S4** Spearman tests for the correlations between α-diversity and the environmental factors.

|  | SL | ML | BL |
| --- | --- | --- | --- |
| Temp | 0.11 | 0.09 | -0.37 *** |
| Salinity | 0.22 * | 0 | -0.03 |
| pH | -0.51 *** | 0.17 | -0.19 |
| DO | -0.18 | 0.36 ** | 0.28 ** |
| NO_2_^-^ | 0.29 ** | -0.37 *** | -0.12 |
| NO_3_^-^ | 0.1 | 0.05 | -0.18 |
| NH_4_^+^ | -0.11 | -0.09 | 0.07 |
| TN | -0.05 | -0.42 *** | -0.21 * |
| TP | 0.33 *** | -0.31 ** | 0.25 * |
| PO_4_^3-^ | -0.14 | 0.41 *** | 0.05 |
| TOC | -0.27 ** | 0.05 | 0.21 * |
| COD | -0.34 *** | -0.27 * | -0.01 |
| Chl-*a* | 0.43 *** | 0.07 | 0.31 *** |

SL: surface layer water; ML: Middle layer water; BL: Bottom layer water.

**Table S5** Mantel tests for the correlations between β-diversity and the distance of individual environmental factors.

|  | SL | ML | BL |
| --- | --- | --- | --- |
| Temp | 0.268*** | 0.487*** | 0.403*** |
| Salinity | 0.392*** | 0.099* | 0.164*** |
| pH | 0.231*** | 0.124* | 0.195*** |
| DO | 0.442*** | 0.588*** | 0.297*** |
| NO_2_^-^ | 0.183** | 0.097 | 0.238*** |
| NO_3_^-^ | -0.039 | -0.005 | 0.216*** |
| NH_4_^+^ | -0.037 | 0.106 | 0.051 |
| TN | -0.059 | 0.093 | 0.387*** |
| TP | 0.143** | 0.153* | 0.059* |
| PO_4_^3-^ | 0.31*** | 0.118* | 0.122** |
| TOC | 0.052 | 0.16* | 0.107** |
| COD | 0.043 | 0.095* | 0.132*** |
| Chl-*a* | 0.065 | 0.06 | 0.159*** |

SL: surface layer water; ML: Middle layer water; BL: Bottom layer water.

**Table S6** Spearman tests for the correlations between community stability, complexity and the environmental factors.

|  | SL | | ML | | BL | |
| --- | --- | --- | --- | --- | --- | --- |
|  | Stability | Complexity | Stability | Complexity | Stability | Complexity |
| Temp | -0.25 ** | -0.1 | 0.12 | -0.04 | -0.29 ** | 0.27 ** |
| Salinity | 0.05 | -0.02 | 0.21 | -0.08 | -0.39 *** | 0.28 ** |
| pH | 0.16 | -0.01 | -0.12 | 0.41 *** | 0.03 | -0.19 |
| DO | 0.23 * | -0.27 ** | 0.29 * | -0.12 | 0.26 * | -0.39 *** |
| NO_2_^-^ | 0 | 0.29 ** | -0.34 ** | 0.53 *** | 0.3 ** | -0.01 |
| NO_3_^-^ | 0.11 | -0.27 ** | -0.28 * | 0.32 ** | -0.11 | -0.39 *** |
| NH_4_^+^ | -0.36 *** | -0.17 | 0.44 *** | 0.24 * | -0.06 | 0.31 ** |
| TN | -0.24 * | 0.09 | -0.08 | 0 | -0.37 *** | -0.09 |
| TP | -0.39 *** | 0.12 | 0.07 | -0.15 | -0.29 ** | -0.05 |
| PO_4_^3-^ | 0.18 | 0.04 | 0 | -0.25 * | -0.08 | 0.14 |
| TOC | 0.37 *** | -0.29 ** | -0.09 | 0.27 * | 0.23 * | -0.18 |
| COD | 0.31 ** | -0.18 | -0.24 * | -0.32 ** | -0.01 | 0.21 * |
| Chl-*a* | -0.23 * | -0.02 | 0.24 * | 0.4 *** | 0.05 | -0.04 |

SL: surface layer water; ML: Middle layer water; BL: Bottom layer water.
